# Evaluation of the content of Zn, Cu, Ni and Pb as well as the enzymatic activity of forest soils exposed to the effect of road traffic pollution

**DOI:** 10.1007/s11356-017-0013-3

**Published:** 2017-09-05

**Authors:** Agata Bartkowiak, Joanna Lemanowicz, Barbara Breza-Boruta

**Affiliations:** 1 Department of Soil Science and Soil Protection, Faculty of Agriculture and Biotechnology, UTP University of Science and Technology, 6 Bernardyńska Street, 85-029 Bydgoszcz, Poland; 2 Sub-Department of Biochemistry, Faculty of Agriculture and Biotechnology, UTP University of Science and Technology, 6 Bernardyńska Street, 85-029 Bydgoszcz, Poland; 3 Department of Microbiology and Food Technology, Faculty of Agriculture and Biotechnology, UTP University of Science and Technology, 6 Bernardyńska Street, 85-029 Bydgoszcz, Poland

**Keywords:** Alkaline and acid phosphatise, Catalase, Dehydrogenases, Heavy metals, Soil, Road traffic pollution

## Abstract

The paper evaluates the contents of total forms of selected heavy metals (Zn, Cu, Ni and Pb) as well as the activity of catalase (CAT), dehydrogenases (DEH), alkaline phosphatase (AlP) and acid phosphatase (AcP) in mineral surface horizons of forest soils exposed to the effect of road traffic pollutions. The sampling locations (*n* = 24) were determined in the area covered by the Szubin Forest along the exit road from Bydgoszcz to Poznań (provincial road no. 223). Soil was sampled 25 m away from the traffic lane, from two depths, 5–20 cm (humus horizons) and 20–50 cm (eluvial horizons). The contents of the heavy metals analysed were in the order of Pb > Zn > Cu > Ni. Despite intensive road traffic, with the Integrated Pollution Index (*IPI*) calculated, there was found a low pollution with nickel, average with zinc and copper and high with lead only. However, under the Regulation of the Minister of Environment, heavy metal values recorded allow for classifying the soils analysed as soils unpolluted with those metals. In the soil samples analysed, there were found significant positive dependencies between the content of clay fraction and zinc (*r* = 0.455; *P* < 0.05) and copper (*r* = 0.430; *P* < 0.05). With the enzyme activity results, values of the soil resistance index (*RS*) were calculated. The enzymes analysed were classified in the following decreasing order in terms of their resistance to traffic pollution: catalase > acid phosphatase > alkaline phosphatase > dehydrogenases (humus horizons) and catalase > dehydrogenases > alkaline phosphatase > acid phosphatase (eluvial horizons). Organic carbon showed a significant positive correlation with the activities of alkaline (*r* = 0.668; *P* < 0.05) and acid phosphatase (*r* = 0.668; *P* < 0.05) however not with catalase and dehydrogenases.

## Introduction

The transformations which occur in the contemporary world together with technology development have resulted in essential changes in the natural environment. A rapid increase in the mobility of people, road transport and motorisation have a deteriorating effect on the condition of the environment (Klimowicz and Melke 2000, Pagotto et al. 2001, Glaser et al. 2005). Transport routes with high traffic intensity are a source of emissions of toxic gases, particulates, and aerosols containing heavy metals. A considerable part of the pollution emitted is deposited on the surface of the land in the vicinity of the source of emissions. The soils adjacent to heavy traffic transport routes are especially exposed to increased amounts of heavy metals (Dzierżanowski and Gawroński 2011). Soils are exposed to pollution with the substances derived from fuel combustion, tire wear and road surface particles as well as chemicals applied in winter to combat black ice. The problem of soil, found in the vicinity of busy roads, with automotive contamination with heavy metals occurs mostly in the districts of big cities with high population density and dense transport network, where a very high number of cars move around and a more difficult road traffic contributes to high emissions of exhaust fumes on a relatively small areas (Binggan and Yang 2010). Heavy metals can be transported into the roadside soils by atmospheric precipitation or road runoff (Vilard et al. 2004, Nabuala et al. 2006). Soil contamination with heavy metals is very rarely observed; however, it demonstrates very dangerous, delayed effects in terms of the environment ecotoxicology. Adsorptive and buffering soil properties make all the heavy metals get strongly accumulated in soil. The content of heavy metals in soil is, to much extent, related to the distance from the roads, traffic intensity and landforms, as well as the method of its use. Monitoring studies have been conducted in many cities and regions in the world to investigate the roadside heavy metal contamination, including China’s Hong Kong (Li et al. 2004), Beijing (Chen et al. 2005; Chen et al. 2010) and Shanghai (Shi et al. 2008), Mexico City (Morton-Bermea et al. 2002), Turkey’s Elazig (Bakirdere and Yaman 2008), England’s Yorkshire (Akbar et al. 2006) and Greece’s Kavala (Christoforidis and Stamatis 2009) and Nigeria (Enuneku et al. 2017) .

Soil contamination with heavy metals has a negative effect on the enzymatic activity. Most heavy metals are indispensable for an adequate functioning of enzymes. Soil enzymes, as natural catalysts of many soil processes connected with decomposition of organic substance, participate in the processes of releasing and making minerals available to plant organisms. The enzymatic activity is an early indicator of changes in the level of intensity of biological processes and the level of soil degradation and it is usually correlated with its physical and chemical properties*.* The soil resistance (*RS*) index for enzymes is highly useful for evaluations of soil quality in some heavy metal-contaminated environments.

Acccording to Alloway (2013), soils in all urban areas are generally contaminated with zinc (Zn), copper (Cu), nickel (Ni) and lead (Pb) from road traffic. These heavy metals accumulate in roadside soils where bioaccumulation by soil fauna and flora may take place with potential negative ecological effects. The aim of this paper was to evaluate the contents of total forms of selected heavy metals (Zn, Cu, Ni and Pb) as well as the enzymatic activity in mineral surface horizons of forest soils in the vicinity of provincial road no. 223. The average road traffic was about 24,000 vehicles daily. These roads experience very high vehicular traffic emissions from long trucks, vehicles and buses. This research project continues the topic of monitoring the study in the area of the Szubin Forest, located in one of the protected areas around Bydgoszcz. This research is an element of the comprehensive woodland protection program and it has served as an attempt of evaluating a potential threat to the environmental safety in the area under study.

## Material and methods

### Location of soil sampling

**Fig. 1:**
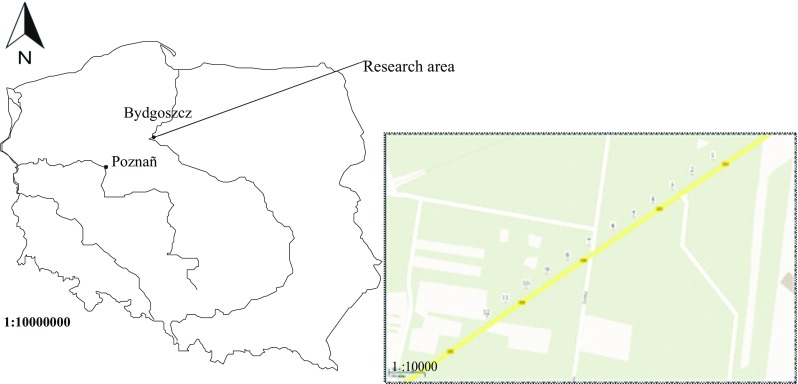
Study area location

The material analysed involved 24 soil samples taken along the route from Bydgoszcz to Poznań (provincial road no. 223) 25 m away from the road located in the Szubin Forest (53 120 N, 18 010 E, central Poland, Europe) (Fig. 1). The local climate is a typical temperate climate with an annual mean temperature of the study area of 8.3 °C. The annual rainfall is about 515 mm. The Szubin Forest is a fresh pine forest located on sandy and sandy-loam soils. The forest species composition is dominated by Scots pine (*Pinus sylvestris*) growing in a fresh coniferous forest habitat (Bśw). The undergrowth is dominated by European blueberry (*Vaccinium myrtillus*) and lily of the valley (*Convallaria majalis*). For the purpose of research, 12 measurement points were determined; the distance between the points was 100 m. Soil was sampled from the mineral horizons of soils from two depths, 5–20 cm (humus horizon) and 20–50 cm (eluvial horizon). Those soils were classified as Albic Podzols (IUSS WRB 2014). The control sample was made up of the soil sampled from the same depths, from the point 1000 m away from the pollution emitter (road no. 223).

### Soil analysis

All the assays in the laboratory were made in three replications. The paper presents the arithmetic mean of the results. Soil samples were collected in September 2014. The soil samples were sampled from each genetic horizon of the soil in three replicates with a soil auger according to the soil sampling procedure of the ISO 10381-2:^2002^. In air-dried soil samples with disturbed structure, sieved through ø 2 mm, some physicochemical properties such as particle size of fraction (%), organic carbon (Corg) and pH were presented in earlier research (Bartkowiak 2015). The total content of heavy metals was determined with Crock and Severson’s method (1980) after the mineralization in the mixture of HF + HClO_4_ acids. The total content forms were determined using the method of atomic absorption spectroscopy with the PU 9100X spectrometer (Philips) and available phosphorus (AP)—with the Egner-Riehm method, DL (PN-R-04023, 1996).

The activity of selected redox enzymes, namely dehydrogenases (DEH) activity [EC 1.1.1] with 2, 3, 5-triphenyl tetrazolium chloride and a measurement of triphenylformazan (TPF) for absorbance at 546 nm, was determined and expressed as mg of TPF kg^−1^ soil 24 h^−1^ according to Thalmann method (1968). The activity of catalase [E.C. 1.11.1.6] (CAT) in soil with the Johnson and Temple method (1964) with 0.3% hydrogen peroxide solution as a substrate was assayed. The residual H_2_O_2_ was determined by titration of 0.02 M KMnO_4_ under acidic conditions. The activity of selected enzymes was also defined, representing the class of hydrolases, alkaline phosphatase [E.C. 3.1.3.1] (AlP) and acid phosphatase [E.C. 3.1.3.2] (AcP), with the method of Tabatabai and Bremner (1969). It is based on the colorimetric assaying of released substrate, p-nitrophenylphosphate (pNP), after the incubation of soil with MUB (modified universal buffer) at pH 6.5 for acid phosphatase and pH 11.0 for alkaline phosphatase samples for 1 h at a temperature of 37 °C.

### Statistical analysis

In order to assess the degree of contamination with heavy metals, it was considered advisable to calculate the Integrated Pollution Index (*IPI*). Then *IPI* was classified into three categories: low (*IPI* ≤ 1), average (1 ≤ *IPI* < 2) and high (*IPI* > 2) (Guo et al. 2012). The *IPI* for each metal was calculated as the ratio of the content of the metal in the surface horizon to its content in the bottom horizon by using the equation (Wei and Yang 2010) *IPI* =$\frac{C}{B}$, where *C*—the mean content of metals from at least 12 sampling sites and *B*—the content of the heavy metals in the bottom horizon (geochemical background). The sum of *IPI* for all the metals studied yields the so-called contamination degree (*C*_deg_) of the ecosystem. In the paper on geochemical background, the following values for Zn—18.0 mg kg^−1^, Cu—4.0 mg kg^−1^, Pb—7.7 mg kg^−1^ and Ni—4.0 mg kg^−1^ were found (Czarnowska 1996).

Based on the enzymatic activities of the samples, the Biological Index of Fertility (BIF) was calculated according to Stefanica et al. (1984),$BIF=\frac{1.5\  DEH+100\  kCAT}{2}$, where *k* is the factor proportionality equal to 0.01.

The resistance index (*RS*) determined according to the activity of selected enzymes to soil was calculated using the formula proposed by Orwin and Wardle (2004), $RS=1-\frac{2\left|D0\right|}{C0+\left|D0\right|}$,where *D*_0_ = *C*_0_—*P*_0_, *C*_0_—parameter value in control (uncontaminated) soil over time *t*_0_, *P*_0_—parameter value in disturbed (contaminated) soil over time *t*_0_. The value of the resistance and resilience index is bounded by − 1 and + 1.

To evaluate the effect of the activity of the enzymes under study, the results were converted and expressed as percentage changes in the activity as compared with the control soil, applying the formula provided by Chaer et al. (2009)$, RCh=\left(\frac{T}{C}-1\right)\times 100$, where *T*—the mean enzyme activity in the treated soil sample and *C*—the mean value obtained for the control.

To identify any potential correlations between soil parameters, the statistical analysis of the results was made using the Statistica software. The correlation matrix studied was based on Pearson’s correlation coefficients using *P* < 0.05 to indicate the 95% probability levels. Basic statistics were used to study tendencies (mean) and the variability (standard deviation (*SD*), coefficient of variation (*CV*), minimum and maximum) of the sample population.

The coefficient of variation of the parameters analysed was calculated as$CV=\left(\frac{SD}{\mathrm{Mean}}\right)\times 100$, where *СV*—coefficient of variation (%), *SD*—standard deviation and mean—arithmetic mean. The values, 0–15%, 16–35% and > 36%, indicate low, moderate or high variability, respectively (Wilding 1985).

## Results and discussion

**Fig. 2:**
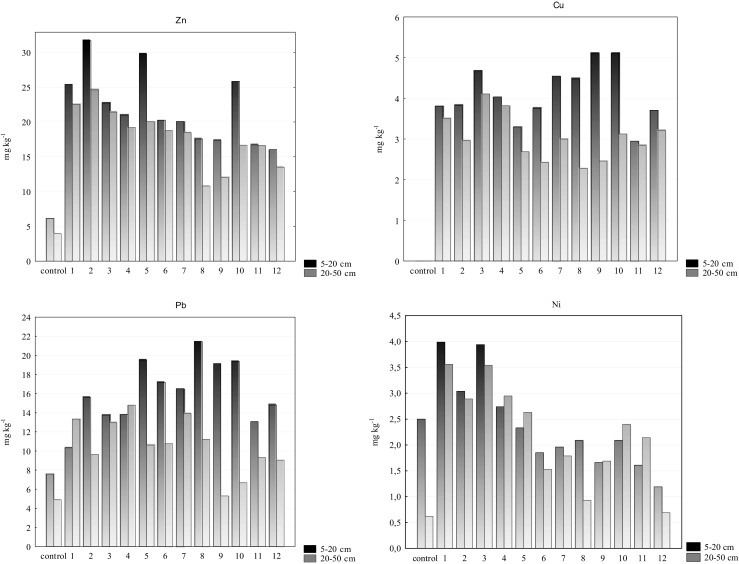
Content of heavy metals in forest soil

The results of the physicochemical parameters of the soil samples have been described in details in the paper by Bartkowiak (2015). Table 1 presents basic properties: reaction, content of organic carbon and the particle size of fraction (%). The available phosphorus content fell in the range of 0.095–29.34 mg kg^−1^ (mean 8.77 mg kg^−1^ for humus horizons) and of 0.158–32.02 mg kg^−1^ (mean 14.55 mg kg^−1^ for eluvial horizons) (Table 1). According to PN-R-04023 (1996), this soil classifies as class V with a very low content of AP. The content of the heavy metals analysed varied and it ranged from 6.15 to 31.72 mg kg^−1^ for Zn, from below the detection limit to 5.11 mg kg^−1^ for Cu, from 1.19 to 3.99 mg kg^−1^ for Ni, from 7.62 to 21.47 mg kg^−1^ for Pb in humus horizons as well as from 3.95 to 24.64 mg kg^−1^ for Zn, from below the detection limit to 4.11 mg kg^−1^ for Cu, from 0.62 to 3.56 mg kg^−1^ for Ni and from 4.91 to 14.81 mg kg^−1^ for Pb in the eluvial horizons (Table 2). The values were lower than reported by other authors both at home and abroad (Akbar et al. 2006, Zhang et al. 2012). The contents of the heavy metals analysed were in the order of Pb > Zn > Cu > Ni. Though the use of unleaded gasoline has caused a subsequent reduction in fuel emissions of Pb, it may still occur in exhaust gas and come from worn metal alloys in the engine (Zhang et al. 2012). The amounts of the metals in the control sample were lower than taken along the route (from point 1 to 12) (Fig. 2). Literature reports on an excessive accumulation of trace elements is limited to about 150 m on both sides of the road and, having exceeded that distance, it assumes the values for unpolluted areas (Nabulo et al. 2006, Zehetner et al. 2009). Higher amounts of the metals studied were found in humus horizons. Heavy metals accumulated in the upper soil horizons demonstrate a high chemical affinity to considerable amounts of organic matter contained in that horizon, which, as a result, slows down its decomposition and decreases the availability (Hernandez et al. 2003). The analysis of correlation has shown dependencies between organic carbon and the content of total forms of lead (*r* = 0.458; *P* < 0.05) (Table 5). Similar dependencies were recorded by Gondek and Filipek-Mazur (2003) investigating the bonding of heavy metals by humus in soils exposed to the effect of road traffic pollution. As seen from scientific literature, the bonding strength for respective heavy metals with organic substance is not the same. A lower adsorption of zinc, copper and nickel by organic substance can be due to a greater chemical affinity of the functional groups of humus acids to lead. Humus compounds play an essential role in the processes of bonding, mobilisation and migration of lead, which can substantially change the mobility of that element in soil. Of all the components of the soil sorption complex, clay minerals are also important for the sorption of heavy metals. In the soil samples analysed, there were found significant positive dependencies between the content of clay fraction in zinc (*r* = 0.455; *P* < 0.05) and in copper (*r* = 0.430; *P* < 0.05).

**Table 1:** Selected physicochemical properties of the soils studied (Bartkowiak 2015)

	Depth (cm)	pH		Corg (g kg^−1^)	AP (mg kg^−1^)	Particle size of fraction (%)		
		H_2_O	KCl			Sand	Silt	Clay
Min	5–20	3.88	3.57	3.05	0.095	76.93	9.35	0.75
	20–50	4.03	3.72	0.60	0.158	78.39	11.08	1.05
Max	5–20	5.19	4.61	17.20	29.34	89.85	22.11	1.79
	20–50	4.98	4.70	8.70	32.02	87.55	19.86	1.96
Mean	5–20	4.41	4.06	9.23	8.773	81.62	17.14	1.24
	20–50	4.49	4.31	4.40	14.55	83.06	15.35	1.57

*AP* Available phosphorus

**Table 2:** Statistical parameters of zinc (Zn), copper (Cu), nickel (Ni), lead (Pb), catalase (CAT), dehydrogenases (DEH), alkaline phosphatase (AlP) and acid (AcP) phosphatase

	Depth (cm)	Zn	Cu	Ni	Pb	CAT	DEH	AlP	AcP
Min	5–20	6.15	b.d.l.	1.19	7.62	0.085	0.065	0.601	0.765
	20–50	3.95	b.d.l.	0.62	4.91	0.072	0.042	0.427	0.430
Max	5–20	31.72	5.11	3.99	21.47	0.111	0.170	1.858	3.212
	20–50	24.64	4.11	3.56	14.81	0.105	0.129	1.428	3.019
Mean	5–20	20.87	3.79	2.38	15.57	0.098	0.104	1.229	1.970
	20–50	16.80	2.80	2.10	10.21	0.089	0.082	0.716	1.110
*CV*	5–20	31.82	34.61	35.84	25.21	8.21	29.51	33.55	36.72
	20–50	33.15	35.63	47.63	31.11	10.14	42.00	42.43	58.83
*SD*	5–20	6.63	1.31	0.85	3.93	0.008	0.030	0.412	0.723
	20–50	5.58	1.00	1.00	3.17	0.009	0.034	0.304	0.653

*b*.*d*.*l*. below detection limit, *CV* coefficient of variation, *SD* standard deviation

The high Pearson correlation coefficient between Zn and Cu (*r* = 0.681; *P* < 0.05) and Pb (*r* = 0.569; *P* < 0.05) and between the total content of Cu and Pb (*r* = 0.738; *P* < 0.05) indicates similar sources of these metals. Those heavy metals are considered as typical metals coming from brake lining abrasion and they are also present together in the rubber used for tire production (Westerlund 2001, Lin et al. 2005).

The Integrated Pollution Index (*IPI*) value calculated in the humus horizons results a low pollution with nickel, average with zinc and copper and high with lead (Table 3). Metal contaminants in soils can have serious implications for the soil ecosystem, soil organisms and human health. It is necessary to evaluate the extent of the risks to potential receptors. The comprehensive evaluation of the state of contamination of the soil under study was made based on the value of the level of contamination (*C*_deg_). The level of contamination ranged from 2.4 to 5.77 which, according to the Håkanson classification (1980), points to a low level of soil contamination with heavy metals. The overall pollution degrees of the heavy metals analysed were in the order of Pb > Zn > Cu > Ni.

**Table 3:** Integrated Pollution Index (*IPI*) and degrees (*C*_*deg*_) of soil contamination

Sampling sites	Depth (cm)	*IPI*				*C*_deg_	*IPI* in *C*_deg_ (%)			
		Zn	Cu	Ni	Pb		Zn	Cu	Ni	Pb
Control point	5–20	0.34	b.d.l.	0.63	0.99	1.96	17.43	0	31.89	50.49
	20–50	0.22	b.d.l.	0.16	0.64	1.02	21.51	0	15.20	62.52
1	5–20	1.41	0.95	1.00	1.34	4.70	30.09	20.27	21.22	28.60
	20–50	1.25	0.88	0.89	1.73	4.75	26.36	18.42	18.74	36.50
2	5–20	1.76	0.96	0.76	2.03	5.51	31.96	17.38	13.79	36.82
	20–50	1.37	0.74	0.72	1.25	4.08	33.55	18.14	17.71	30.75
3	5–20	1.26	1.17	0.99	1.79	5.21	24.27	22.46	18.91	34.35
	20–50	1.19	1.03	0.89	1.69	4.80	24.81	21.41	18.44	35.12
4	5–20	1.17	1.01	0.69	1.80	4.67	24.98	21.63	14.67	38.46
	20–50	1.07	0.96	0.74	1.92	4.69	22.78	20.36	15.72	41.01
5	5–20	1.66	0.83	0.58	2.54	5.61	29.52	14.71	10.38	45.23
	20–50	1.12	0.67	0.66	1.38	3.81	29.28	17.65	17.26	36.23
6	5–20	1.13	0.94	0.46	2.22	4.76	23.68	19.75	9.72	46.93
	20–50	1.04	0.61	0.38	1.40	3.43	30.39	17.71	11.15	40.82
7	5–20	1.12	1.14	0.49	2.15	4.90	22.78	23.21	10.00	43.84
	20–50	1.03	0.75	0.45	1.81	4.04	25.40	18.56	11.08	44.78
8	5–20	0.98	1.12	0.52	2.79	5.41	18.05	20.70	9.66	51.54
	20–50	0.60	0.57	0.23	1.46	2.86	21.04	19.93	8.13	51.04
9	5–20	0.97	1.28	0.42	2.49	5.16	18.77	24.71	8.04	48.25
	20–50	0.67	0.62	0.42	0.69	2.40	27.87	25.63	17.60	28.73
10	5–20	1.44	1.28	0.52	2.52	5.76	24.95	22.18	9.07	43.72
	20–50	0.93	0.78	0.60	0.87	3.18	29.18	24.61	18.87	27.32
11	5–20	0.93	0.74	0.40	1.70	3.77	24.65	19.56	10.68	45.13
	20–50	0.92	0.71	0.54	1.21	3.38	27.28	21.08	15.83	35.81
12	5–20	0.89	0.93	0.30	1.93	4.05	22.02	22.90	7.35	47.59
	20–50	0.75	0.80	0.17	1.18	2.90	25.88	27.67	5.95	40.57

*b*.*d*.*l*. below detection limit, *IPI* Integrated Pollution Index, *IPI* in *C*_deg_ degree of contamination

Classifying with the grain size composition the soils under study are considered “very light”, according to the criteria assumed, they can be referred to as 0 degree, covering the soils unpolluted with heavy metals (Terelak et al. 2000). Under the Regulation of the Minister of Environment dated 9 September 2002 on soil quality and land quality standards (Dz. U. No. 165, item. 1359, 2002) and on assessment procedures for the land surface pollution (Dz. U. item. 1395, 2016), the values of the heavy metals under study classify the soils analysed as unpolluted soils. All soil samples had lower values of total heavy metal content than those recommended by the U.S. Environmental Protection Agency (1993). The variation in the content of Zn, Cu, Ni and Pb may be the result of decreased atmospheric deposition from road traffic in the soil and the contribution of meteorological factors.

**Fig. 3:**
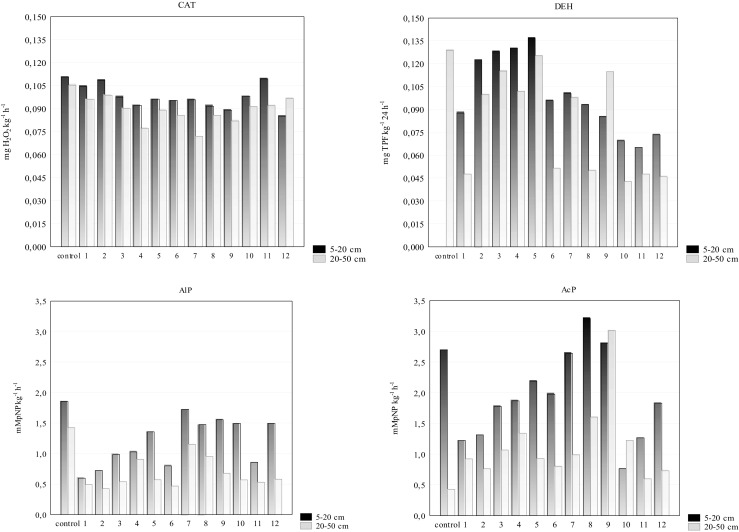
Activity of the catalase (CAT), dehydrogenases (DEH), alkaline phosphatase (AlP) and acid phosphatase (AcP)

The intensity and tendency of variations depended on the type of enzyme under study and the soil-sampling depth (Fig. 3). The analysis of soil enzymatic activities (CAT, DEH, AlP, AcP) showed the lower soil enzyme activities in the soil along the major roads than in the control sample.

The activity of soil catalase in the humus horizons ranged from 0.085 to 0.110 mg H_2_O_2_ kg^−1^ h^−1^ (mean 0.097 mg H_2_O_2_ kg^−1^ min^−1^) (Fig. 2). With the *RCh* index calculated, it was found that the CAT activity decreased by an average of 12.37% (1.92–23.0%) as compared with the control soil (Table 4). The activity of dehydrogenases in the humus horizons decreased by an average of 0 41.65%, whereas in the eluvial horizons—by an average of 39.08% as compared with the control soil.

**Table 4:** Biological Index of Fertility (*BIF*), resistance (*RS*) of soil enzymes and relative changes (*RCh*) of activity in roadside soils

Sampling sites	Depth (cm)	*BIF*	*RS*				*RCh* (%)			
			CAT	DEH	AlP	AcP	CAT	DEH	AlP	AcP
Control point	5–20	0.183	–	–	–	–	–	–	–	–
	20–50	0.150	–	–	–	–	–	–	–	–
1	5–20	0.118	0.895	0.349	0.193	0.293	− 5.50	− 48.3	−67.6	− 54.7
	20–50	0.084	0.839	0.228	0.210	− 0.072	− 8.74	− 62.8	−65.2	115
2	5–20	0.147	0.962	0.566	0.243	0.322	− 1.90	− 27.7	−61.0	− 51.3
	20–50	0.124	0.883	0.632	0.176	0.127	− 6.24	− 22.5	− 70.1	77.5
3	5–20	0.145	0.788	0.604	0.362	0.492	− 11.9	− 24.7	− 46.8	− 34.1
	20–50	0.132	0.747	0.809	0.233	− 0.196	− 14.50	− 10.56	− 62.2	148
4	5–20	0.144	0.714	0.623	0.384	0.530	− 16.7	− 23.2	− 44.6	− 30.7
	20–50	0.115	0.578	0.655	0.466	− 0.360	− 26.78	− 20.83	− 36.5	212
5	5–20	0.151	0.769	0.673	0.577	0.682	− 13.0	− 19.6	− 26.8	− 18.9
	20–50	0.139	0.732	0.947	0.250	− 0.072	− 15.50	− 2.71	− 60.0	115
6	5–20	0.120	0.757	0.394	0.274	0.579	− 19.8	− 43.5	− 57.0	− 26.7
	20–50	0.082	0.685	0.251	0.197	0.077	− 18.72	− 59.8	− 67.1	85.6
7	5–20	0.124	0.763	0.421	0.871	0.957	− 13.4	− 40.7	− 6.90	− 2.2
	20–50	0.110	0.520	0.611	0.677	− 0.132	− 31.61	− 24.13	− 19.3	130
8	5–20	0.116	0.706	0.379	0.661	0.683	− 17.3	− 45.1	− 20.4	18.9
	20–50	0.080	0.685	0.240	0.501	− 0.465	− 18.72	− 61.24	− 33.3	273
9	5–20	0.109	0.673	0.335	0.722	0.921	− 19.6	− 49.8	− 16.1	7.10
	20–50	0.127	0.637	0.804	0.313	− 0.715	− 22.1	− 10.8	− 52.3	601
10	5–20	0.101	0.797	0.258	0.671	0.165	− 11.3	− 59.0	19.6	− 71.7
	20–50	0.078	0.765	0.199	0.249	− 0.299	− 13.29	− 66.7	− 60.1	185
11	5–20	0.104	0.977	0.238	0.301	0.307	− 1.20	− 61.5	− 53.7	53.80
	20–50	0.082	0.775	0.228	0.229	− 0.431	− 12.68	− 62.89	− 62.8	39.8
12	5–20	0.098	0.626	0.276	0.675	0.514	− 23.0	− 59.7	− 19.4	− 32.1
	20–50	0.083	0.848	0.217	0.257	0.178	− 8.25	− 64.3	− 59.2	69.8

The activities of alkaline phosphatase in humus horizons soil ranged from 0.601 to 1.858 mM pNP kg^−1^ h^−1^ (Fig. 3) (with a mean value of 1.229 mM pNP kg^−1^ h^−1^) and in humus horizons soil from 0.427 to 1.428 mM pNP kg^−1^ h^−1^ (with a mean value of 0.716 mM pNP kg^−1^ h^−1^). According to the Wilding (1985) classification, the CV values of soil catalase activities (8.21% for humus horizons; 10.14 for eluvial horizons) and catalase activity (106%) were low, while the CV values of soil dehydrogenases activities were moderate (29.51% for humus horizons) and high (42.00% for eluvial horizons) (Table 2). Organic carbon (Corg) gave the significant positive correlations with alkaline (*r* = 0.668; *P* < 0.05) and acid phosphatase (*r* = 0.652; *P* < 0.05) (Table 5). Organic matter plays a protective function towards enzymes which thus get immobilised. It has a positive effect on the stability of protein structure, decreasing the sensitivity to negative changes triggered by environmental factors.

**Table 5:** Relationship between selected soil properties (*n* = 24; *P < 0.05*)

Variables		Equation	*r*	*P*
Dependent	Independent			
Fraction < 0.002	Total zinc	*y* = − 0.103 + 0.8861*x*	0.455	0.0196
Fraction < 0.002	Total copper	*y* = − 0.0606 + 0.6057*x*	0.430	0.0282
Fraction < 0.002	Alkaline phosphatase	y = 0.1153 − 0.6952*x*	− 0.641	0.0004
Fraction < 0.002	Available phosphorus	*y* = 0.748 + 0.8334*x*	0.388	0.0498
Fraction < 0.002	Dehydrogenases	*y* = 0.1686 − 1.0168*x*	− 0.408	0.0384
pH KCl	Total zinc	*y* = − 0.0921 + 0.9704*x*	0.644	0.0027
pH KCl	Alkaline phosphatase	*y* = 0.1545 − 0.892*x*	− 0.710	0.00005
Organic carbon	Total lead	*y* = − 0.0868 + 0.847*x*	0.458	0.0185
Organic carbon	Alkaline phosphatase	*y* = 0.0345 + 0.8627*x*	0.668	0.0002
Organic carbon	Acid phosphatase	*y* = − 0.0736 + 1.1064*x*	0.652	0.0003
Total zinc	Total copper	*y* = 0.1622 + 0.2041*x*	0.681	0.0001
Total copper	Total lead	*y* = 0.1034 + 0.6184*x*	0.569	0.00002
*RS*_AlP_	Organic carbon	*y* = 0.174 + 0.0288*x*	0.523	0.0063
*RS*_CAT_	Total zinc	*y* = 0.195 + 0.0266*x*	0.731	0.00002
*RS*_AlP_	Total copper	*y* = − 0.020 + 0.1191*x*	0.648	0.0003

With the results of the activity of catalase and dehydrogenases, the value of the biological index of soil fertility (*BIF*) was calculated (Table 4). Its values were lower both in the humus horizon and in the eluvial horizon as compared with the control soil (0.183 for humus horizons and 0.150 for eluvial horizons). The *BIF* values indicating that index is unable to determine the impact of traffic pollution on soil biological activity.

Decreased values of the soil resistance index (*RS*) showed negative changes in the activity of the enzymes of forest soils along the road traffic route. Soil resistance indicators (*RS*) are effective measures of enzyme responses to environmental stress (Borowik et al. 2014). The values of the soil resistance index (*RS*) calculated for catalase, dehydrogenases and alkaline phosphatase were positive. Orwin and Wardle (2004) report on the *RS* values falling in the range from 1 to − 1, where 1 stands for no effect of human pressure on the parameter studied. One can, therefore, state that the road traffic affected the activity of catalase to a little extent since the *RS* values were from 0.626 to 0.977 in the humus horizons (mean of 0.786) as well as 0.520–0.883 (mean of 0.727) in the eluvial horizons of soil (Table 4). A lower *RS* value was noted for dehydrogenases both in the humus horizon (0.238–0.673; mean 0.426) and in the eluvial horizon (0.199–0.947; mean of 0.485). The value of the soil resistance index calculated for alkaline phosphatase was similar as for dehydrogenases, whereas the *RS* value calculated for acid phosphatase activity in most soils from the eluvial horizons was negative, which points to a very low resistance of that enzyme to the effect of human pressure. Based on the data presented in Table 4 the enzymes were arranged in the following decreasing order in terms of their resistance in the surface horizon: CAT > AcP > AlP > DEH and in the eluvial horizon: CAT > DEH > AlP > AcP. Positive correlations were received between *RS*_*AlP*_ and organic carbon (*r* = 0.523; *P* < 0.05). Organic matter provides a better soil environmental condition for stabilising and protecting extracellular enzymes. Higher organic carbon levels support greater microbial biomass and thus show more enzymatic activity. Significant positive correlations were observed between *RS*_*CAT*_ values and the content of zinc (*r* = 0.730; *P* < 0.05), *RS*_*AlP*_ and cooper (*r* = 0.648; *P* < 0.05). It could have been due to favourable physicochemical properties of the soils, which resulted in a decreased mobility of heavy metals, thus, decreasing their toxicity to enzymatic proteins. A lack of inhibition of the enzymes as affected by heavy metals could have been due to the content of organic matter and clay fraction which bind enzymatic protein, protecting it from negative environmental factors (Doelman and Haanstra 1984). It is also known that heavy metals at little concentrations are activators of many enzymes.

## Conclusion

The contents of the heavy metals analysed were in the order of Pb > Zn > Cu > Ni. There was found a decrease in the total content of the heavy metals with soil sampling depth. The Integrated Pollution Index (*IPI*) values calculated in surface horizons results a high pollution with lead, average with zinc and copper and low with nickel. However, in the area under study, the concentration of heavy metals in soil do not exceed the norms provided by the Regulation of the Minister of Environment and the U.S. Environmental Protection Agency and thus, the soil samples analysed can be considered unpolluted.

Catalase, dehydrogenases and phosphatases activities could be used as indicators for soil contaminated by heavy metals. The results of the activity of some enzymes in soil show the decrease in catalase, dehydrogenase, alkaline and acid phosphatase activities along the major roadside.

The present results demonstrate that forest soils along the high road traffic intensity route do not accumulate hazardous amounts of the heavy metals under study in the surface and subsurface horizons. As for the enzymatic activity, a negative effect of road traffic pollution 25 m away from the traffic lane was identified.
